# Preemptively and non-preemptively transplanted patients show a comparable hypercoagulable state prior to kidney transplantation compared to living kidney donors

**DOI:** 10.1371/journal.pone.0200537

**Published:** 2018-07-16

**Authors:** Gertrude J. Nieuwenhuijs-Moeke, Tamar A. J. van den Berg, Stephan J. L. Bakker, Marius C. van den Heuvel, Michel M. R. F. Struys, Ton Lisman, Robert A. Pol

**Affiliations:** 1 Department of Anaesthesiology, University of Groningen, University Medical Centre Groningen, Groningen, the Netherlands; 2 Department of Surgery, University of Groningen, University Medical Centre Groningen, Groningen, the Netherlands; 3 Department of Nephrology, University of Groningen, University Medical Centre Groningen, Groningen, the Netherlands; 4 Department of Pathology, University of Groningen, University Medical Centre Groningen, Groningen, the Netherlands; 5 Department of Anesthesia, Ghent University, Ghent, Belgium; Public Library of Science, UNITED KINGDOM

## Abstract

To prevent renal graft thrombosis in kidney transplantation, centres use different perioperative anticoagulant strategies, based on various risk factors. In our centre, patients transplanted preemptively are considered at increased risk of renal graft thrombosis compared to patients who are dialysis-dependent at time of transplantation. Therefore these patients are given a single dose of 5000 IU unfractionated heparin intraoperatively before clamping of the vessels. We questioned whether there is a difference in haemostatic state between preemptively and non-preemptively transplanted patients and whether the distinction in intraoperative heparin administration used in our center is justified. For this analysis, citrate samples of patients participating in the VAPOR-1 trial were used and several haemostatic and fibrinolytic parameters were measured in 29 preemptively and 28 non-preemptively transplanted patients and compared to 37 living kidney donors. Sample points were: induction anaesthesia (T1), 5 minutes after reperfusion (T2) and 2 hours postoperative (T3). At T1, recipient groups showed comparable elevated levels of platelet factor 4 (PF4, indicating platelet activation), prothrombin fragment F1+2 and D-dimer (indicating coagulation activation) and Von Willebrand Factor (indicating endothelial activation) compared to the donors. The Clot Lysis Time (CLT, a measure of fibrinolytic potential) was prolonged in both recipient groups compared to the donors. At T3, F1+2, PF4 and CLT were higher in non-preemptively transplanted recipients compared to preemptively transplanted recipients. Compared to donors, non-preemptive recipients showed a prolonged CLT, but comparable levels of PF4 and D-dimer. In conclusion pre-transplantation, preemptively and non-preemptively transplanted patients show a comparable enhanced haemostatic state. A distinction in intraoperative heparin administration between preemptive and non-preemptive transplantation does not seem justified.

## Introduction

Renal artery or vein thrombosis is still one of the most dreaded complications after kidney transplantation. Although the incidence is found between 0.2–7.5% and 0.1–8.2% respectively, it is responsible for up to 45% of early graft loss [1–3]. Graft thrombosis is characterized by (sudden) anuria and, in case of venous thrombosis, pain and or swelling in the iliac fossa. Ojo and colleagues report cumulative frequencies of renal vein thrombosis (RVT) of 16% within the first 24 hours, with cumulative frequencies rising to 62% and 89% on day 10 and 20 post-operative [4]. Although early recognition and surgical intervention may save the graft, it frequently leads to graft loss. As international guidelines are lacking, different intra- and postoperative antithrombotic strategies are used among centres, ranging from no anti-coagulation therapy to unfractionated heparin (UFH) for several days post- transplantation in high risk patients. Known risk factors are donor age <6 or >60 years, recipient age <5 or >50 years, cold ischemia time>24 h, renal atherosclerosis in donor and recipient, donation of the right kidney, peritoneal dialysis, history of diabetes mellitus or thrombosis in recipient, technical difficulties or hemodynamic instability during transplantation, and delayed graft function [1]. The wide incidence range reported is probably due to differences in study populations with highest incidences reported in paediatric kidney transplantation and lowest in studies involving living donor kidney transplantation (LDKT)[5–7]. In LDKT a high proportion of recipients is transplanted preemptively. This is in contrast to transplantation with kidneys from deceased donors where most patients are already dialysis-dependent at time of transplantation. In our centre, patients undergoing preemptive transplantation do, and dialysis-dependent (non-preemptive) patients do not receive intraoperative anticoagulation with the use of a single dose of 5000 IU UFH during kidney transplantation. This difference can be explained by the presumed bleeding risk of dialysis-dependent recipients. Historically, these patients were considered hypocoagulable, because of the residual effect of heparin used during dialysis and the continuous activation of platelets through contact with the dialysis membrane [8]. Recent insights however, suggest that these dialysis dependant patients are at risk of both bleeding and thrombotic complications [9]. We therefore questioned whether there is a difference in haemostatic state between preemptively and non-preemptively transplanted patients and whether the distinction in intraoperative heparin administration used in our center is justified. We compared functional haemostatic tests and markers of in vivo activation of haemostasis between preemptively and non-preemptively transplanted patients before and after kidney transplantation. Results were compared with parameters in their living kidney donors undergoing laparoscopic donor nephrectomy.

## Materials and methods

### Study population

Stored citrated plasma samples of donors and recipients participating in the Volatile Anesthetic Protection Of Renal transplants (VAPOR)-1 trial were used. The VAPOR-1 trial is a prospective randomized controlled trial on the effects of two different anesthetic agents (propofol vs sevoflurane) on renal outcome in LDKT. The Institutional Review Board of the University Medical Center of Groningen approved the study protocol of VAPOR-1 (METc 2009/334), which was conducted in adherence to the Declaration of Helsinki and registered with ClinicalTrials.gov: NCT01248871. Details of this trial have been published previously [10]. Sixty donor and recipient couples met the inclusion criteria and gave written informed consent. Three couples were excluded due to violation of the surgical or immunosuppressive protocol, leaving 57 couples for analysis. Of these 57 recipients 28 patients were transplanted preemptively (preemptive group, PG) and 29 patients were transplanted non-preemptively (non-preemptive, dialysis group, DG). In order to establish reference values for the various test performed we selected 37 patients out of the pool of 57 donors to function as a control group (CG).

### Dialysis, anaesthesia and surgery

Last dialysis was performed the day before surgery in case of haemodialysis (HD) or until one hour before surgery in case of peritoneal dialysis (PD). Kidney donation was performed via hand-assisted laparoscopy. After procurement, the kidney was flushed and perfused with cold University of Wisconsin solution (ViaSpan, DuPont, Wilmington, NC, USA; Belzer UW, Bridge to life, Columbia SC, USA) and placed in cold storage. Kidney transplantation was performed according to the local protocol. Choice of anaesthetic agent (propofol or sevoflurane) was based upon randomization. In all patients, analgesia was managed with remifentanil with the use of target controlled infusion (TCI, Minto [11]). Depth of anaesthesia, administration of fluids, haemodynamic management and the administration of all medications were strictly protocolised. Patients transplanted preemptively were given 5000 IU of UFH before clamping of the external iliac artery according to local protocol.

### Samples

**Fig 1:**
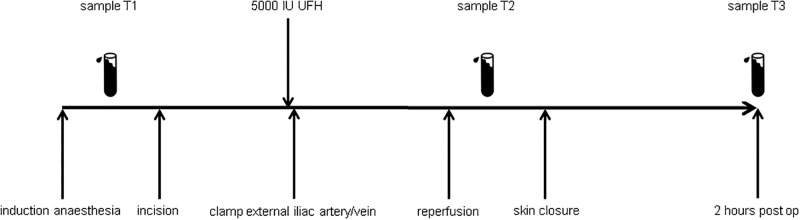
Timeline of the transplantation procedure, blood sampling and administration of heparin. T1: induction of anaesthesia; T2: 5 minutes after reperfusion of the kidney; T3: 2 hours post-operative.

Citrated blood samples were taken at standardized time points. Samples were centrifuged (1500g, 20 min) and stored at -80°C until analysis. For this project we analysed samples taken at three time points (T1-T3): T1; baseline sample at induction of anaesthesia, T2; 5 minutes after reperfusion of the kidney (only recipients) and T3; 2 hours after skin closure (Fig 1). The following haemostatic and fibrinolytic parameters were analysed: Platelet factor 4 (PF4) and soluble platelet selectin (sP-selectin) as marker of platelet activation; Prothrombin fragment 1+2 (F1+2) and D-dimer as marker of coagulation activation; Von Willebrand Factor (VWF) as marker of endothelial activation; Plasma coagulation and fibrinolytic potential was studied by respectively thrombin generation (TGA) and clot lysis time (CLT) assays. At T2, TGA and CLT were not performed due to presence of heparin in samples of the preemptive group, preventing clot formation. Two open needle biopsies from the transplanted kidney were obtained, one prior to implantation and one 30 minutes after reperfusion. Each biopsy was divided into two parts. One part was embedded in paraffin and the other was stored in RNAlater.

### Assays

Platelet factor 4 (PF4) and soluble P-selectin were assessed using a commercially available enzyme-linked immunosorbent assays (ELISA; Duoset, R&D systems, Abingdon, UK). Prothrombin fragment F1+2 was measured with a commercially available ELISA (Siemens Healthcare Diagnostics, Breda, The Netherlands). D-dimer levels were measured on an ACL300 coagulation analyzer using reagents from the manufacturer (Werfen, Breda, The Netherlands). Plasma levels of VWF were determined with an in-house ELISA using commercially available polyclonal antibodies (DAKO, Glostrup, Denmark).

Thrombin generation tests were performed using platelet-poor plasma with the fluorimetric method described by Hemker, Calibrated Automated Thrombography^®^ according to the instructions of the manufacturer [12]. Coagulation was activated using a commercial trigger composed of recombinant tissue factor at a concentration of 5 pM and phospholipids at a concentration of 4 μM (Thrombinoscope BV, Maastricht, The Netherlands) in the presence of a soluble form of thrombomodulin. All experiments were performed in triplicate. The lagtime, endogenous thrombin potential (ETP), peak height, and velocity index were derived from the thrombin generation curves by the Thrombinoscope software.

Fibrinolytic potential was assessed using a plasma-based clot lysis assay. Lysis of a tissue factor–induced clot by exogenous tissue plasminogen activator (tPA) was determined by monitoring changes in turbidity during clot formation and subsequent lysis as described previously [13]. Clot lysis times were derived from the clot-lysis turbidity profiles using in house-generated software. Clot lysis time was defined as the time from the midpoint of the clear to maximum turbid transition, representing clot formation, to the midpoint of the maximum turbid to clear transition, representing the lysis of the clot.

### Pathology

Paraffin embedded reperfusion biopsies were stained with the Martius Scarlet Blue (MSB) staining in order to identify fibrin depositions in the biopsies. After deparaffinization, slides were stained with haematoxylin followed by staining with Martius Yellow solution and Brilliant Crystal Scarlet 6R solution. The staining was performed by placing the slides in a phosphor wolfram acid solution to stain fibrin red, followed by placing the slides in a anilin blue solution to stain collagen blue. After rinsing with acetic acid slides were dehydrated.

### Statistical methods and analyses

Data were analysed with the use of SPSS version 22 (IBM Corp, Armonk, NY, USA) and GraphPad Prism version 5.04 (GraphPad software,Inc, La Jolla, CA, USA). Differences in categorical data were assessed with the use of Fishers’ Exact test or Chi-squared test. Continuous data were tested for normality with the use of the Shapiro-Wilk test. In case of normality ANOVA or t-test were used, if not Kruskal-Wallis test or Mann-Whitney U test was applied. When differences between groups were significant, posthoc testing with the use of Bonferonni was performed. To compare matched data, paired t-test or Wilcoxon matched pairs signed rank test was used. Correlations were tested with the use of Pearson r correlation. Values are given as mean with standard deviation (SD) or median with interquartile range (IQR). Statistical significance was set at *P*< 0.05.

## Results

Baseline characteristics and relevant intraoperative parameters are listed in Table 1. Age, BMI and gender are comparable between groups. Charlson Comorbidity Index (CCI) was scored for donors and recipients. As expected, donors showed a lower CCI compared to recipients. CCI in recipients was comparable. None of the donors was treated with platelet aggregation inhibitors or erythropoietin analogs. The use of these drugs was comparable between preemptive and non-preemptive recipients. Antiplatelet therapy was continued during surgery. None of the patients was treated with vitamin K antagonists. Low Molecular Weight Heparin (LMWH) in prophylactic dose was administered to all donors the evening post donation and to recipients the day after transplantation. There was no difference in the history of thromboembolic events or known predisposing factors for bleeding or thrombosis between preemptively and non-preemptively transplanted patients. Haemoglobin (Hb) level, platelet counts and urea were routinely measured the day before surgery. Donors showed higher Hb levels compared to all recipients and Hb levels of the non-preemptive group were higher compared to the preemptive group. Platelet counts of donors were higher compared to the non-preemptive group. Urea levels of recipients were elevated compared to donors as expected. Preemptively transplanted patients showed higher urea levels compared to non-preemptively transplanted patients, which can be explained by dialysis in the last group. There was no correlation between the level of urea and the haemostatic and fibrinolytic parameters measured with the exception of D-dimers (r 0.522, S1 Table). The estimated glomerular filtration rate (eGFR) of preemptively transplanted patients was higher than the eGFR of non-preemptively transplanted patients. Of the non-preemptively transplanted patients, 21 (72%) patients were on HD, and 8 (28%) on PD. Duration of the laparoscopic donor nephrectomy was longer than the kidney transplantation procedure. Ischemia times were comparable between recipients and the amount of fluid given intraoperatively was comparable between all groups. None of the recipients experienced graft thrombosis, 1 patient in the non-preemptive group experienced a postoperative bleeding complication warranting surgical exploration.

**Table 1:** Baseline characteristics and intraoperative parameters. Data are given as mean (SD) and median (IQR) or n (%). Fishers’ Exact test or Chi-squared test were used in case of categorical data. Continuous data were tested with ANOVA/ Kruskal-Wallis in case of three groups and with student t-test/Mann-Whitney in case of 2 groups.

	Preemptively transplanted PG	Non-preemptively transplanted DG	Living donors CG	*P-value*			
				ANOVA / KW / X^2^	PGvsDG	PGvsCG	DGvsCG
**Baseline characteristics**							
	**n = 28**	**n = 29**	**n = 37**				
**Age years**	50.7 (11.3)	50.9 (13.5)	53.5 (11.2)	0.559	1.000	1.000	1.000
**Gender male n (%)**	11 (40)	16 (55)	17 (46)	0.490	0.710	1.000	1.000
**BMI**	25.1 (3.2)	25.7 (3.9)	26.7 (3.1)	0.145	1.000	0.167	0.670
**Renal disease n (%)**							
**Diabetes mellitus**	5 (18)	0 (0)	N/A		0.024		
**IgA nephropathy**	4 (14)	3 (10)			0.706		
**AIN**	1 (4)	3 (10)			>0.999		
**Glomerulonephritis**	2 (7)	2 (7)			>0.999		
**Vasculitis**	1 (4	2 (7)			0.612		
**PKD**	5 (18	3 (10			0.730		
**Renal atrophy**	3 (11)	5 (17)			>0.999		
**Sclerosis**	4 (14	3 (10)			0.263		
**TIN**	2 (7)	1 (3)			0.194		
**Other**	1 (4)	7 (24)			0.058		
**CCI**	4 (3–5.75)	5 (3–6)	0 (0–0)	<0.001	0.734	<0.001	<0.001
**Platelet aggregation inhibitors n (%)**	6 (21.4)	11 (37.9)	N/A		0.146	N/A	N/A
**Haemoglobin mmol L**^**-1**^	7.3 (0.7)	7.9 (0.8)	8.9 (0.7)	<0.001	0.006	<0.001	<0.001
**Platelet count x10**^**9**^ **L**^**-1**^	228 (62)	201 (75)	248 (64)	0.017	0.350	0.710	0.013
**eGFR ml min**^**-1**^	9.0 (2.8)	7.1 (3.0)	110 (96–129)	<0.001	0.021	<0.001	<0.001
**Urea mmol L**^**-1**^	28.8 (6.6)	20.7 (5.3)	5.7 (5.0–6.2)	<0.001	<0.001	<0.001	<0.001
**Type of dialysis n (%)**	N/A		N/A				
**Haemodialysis**		21 (72)					
**Peritoneal dialysis**		8 (28)					
**Tromboembolic history n (%)**							
**VTE**	3 (11)	4 (14)	N/A	N/A	>0.999	N/A	N/A
**CVA**	1 (4)	3 (10)			0.612		
**SLE**	1 (4)	0 (0)			0.491		
**APS**	0 (0)	1 (3)			>0.999		
**Intraoperative parameters**							
**Duration min**	209 (35)	204 (25)	241 (37)	<0.001	0.425	0.002	<0.001
**Ischemia times (min)**			N/A			N/A	N/A
**WIT1**	4.1 (0.7)	3.7 (1.5)			0.269		
**CIT**	172 (24)	179 (36)			0.427		
**WIT2**	43 (7)	43 (7)			0.915		
**Anesthetic agent n (%)**							
**Propofol**	9 (32)	9 (31)	26 (70)	0.001	0.929	0.002	0.002
**Sevoflurane**	19 (68)	20 (69)	11 (30)				

PG; preemptive group; DG: non-preemptive dialysis group; CG: living donor control group; KW: Kruskal- Wallis; X^2^: Chi-squared. n: number in group; BMI: body mass Index; AIN: auto immune nephropathy; PKD: Polycystic Kidney Disease;TIN: tubulo interstitial nefritis; CCI: Charlson Comorbidity Index; eGFR: estimated glomerular filtration rate; VTE: venous thromboembolism; CVA: cerebro vascular attack; SLE: systemic lupus erythematodes; APS: antiphospholipid syndrome; WIT1: Warm Ischemia Time 1; CIT: Cold Ischemia Time defined as the total cold storage time; WIT2: Warm Ischemia Time 2 defined as the time between cold storage and recirculation (anastomosis time).

### Platelet activation

**Fig 2:**
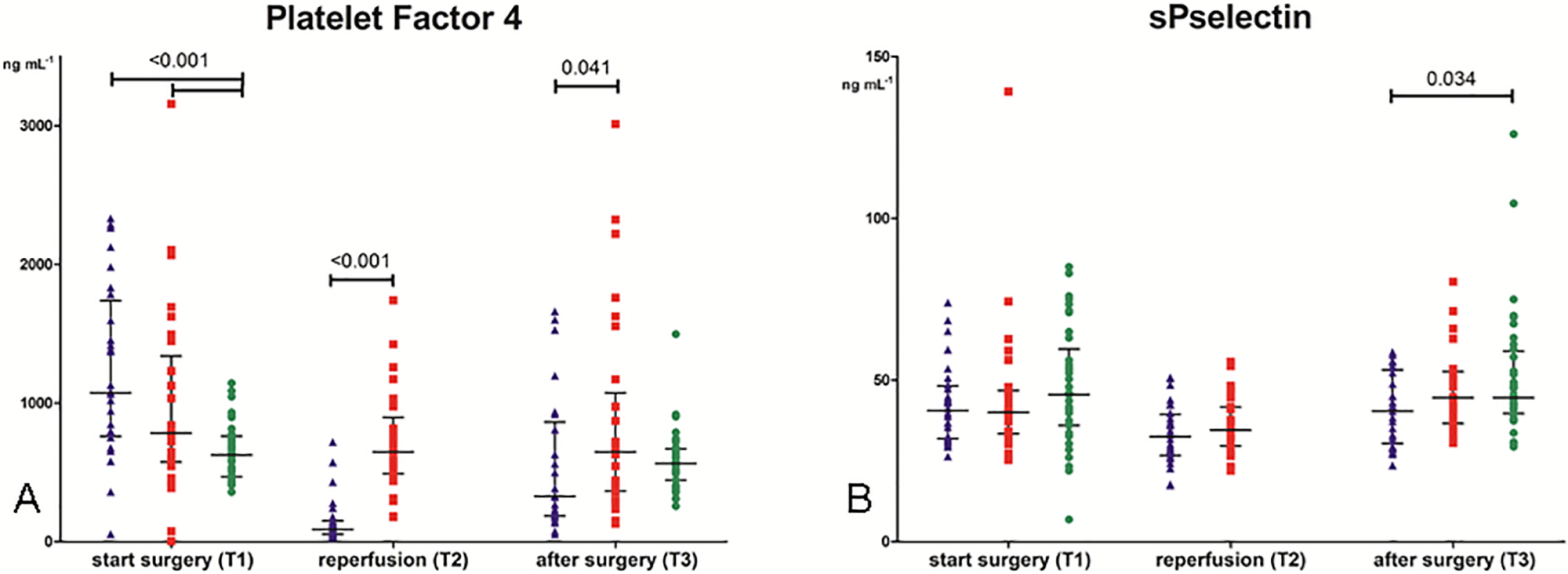
Markers of platelet activation. Fig 2A: Levels of platelet factor 4. Preemptive group (blue triangle), non-preemptive group (red square) and donors (green dot) before incision (T1), 5 minutes after reperfusion (T2) and 2 hours after surgery (T3). Data are given as medians with IQR. Fig 2B: Levels of soluble P-selectin. Preemptive group (blue triangle), non-preemptive group (red square) and donors (green dot) before incision (T1), 5 minutes after reperfusion (T2) and 2 hours after surgery (T3). Data are given as medians with IQR.

Levels of platelet activation markers are shown in Fig 2A (PF4) and Fig 2B (sP-selectin). At baseline (T1), PF4 levels were comparable between the preemptive and non-preemptively transplanted patients but both groups showed higher levels compared to donors (1074 (762–1739) and 784 (575–1340) *versus* 625 (469–763) ng mL^-1^; P<0.001). Five minutes after reperfusion (T2), levels in the non-preemptive group were higher compared to the preemptive group (648 (492–897) *versus* 89 (56–151) ng mL^-1^; P<0.001). Two hours post-operative (T3), PF4 levels were higher in the non-preemptive group compared to the preemptive group (647 (366–1074) *versus* 328 (187–865) ng mL^-1^; P = 0.041), but levels were comparable to the donors (564 (444–671)).

Levels of sPselectin were comparable between groups at each sample point, with exception of lower levels of sPselectin in the preemptive group compared to the donors at T3 (40 (30–53) versus 45 (40–59) ng/mL, P = 0.034).

### Coagulation activation

**Fig 3:**
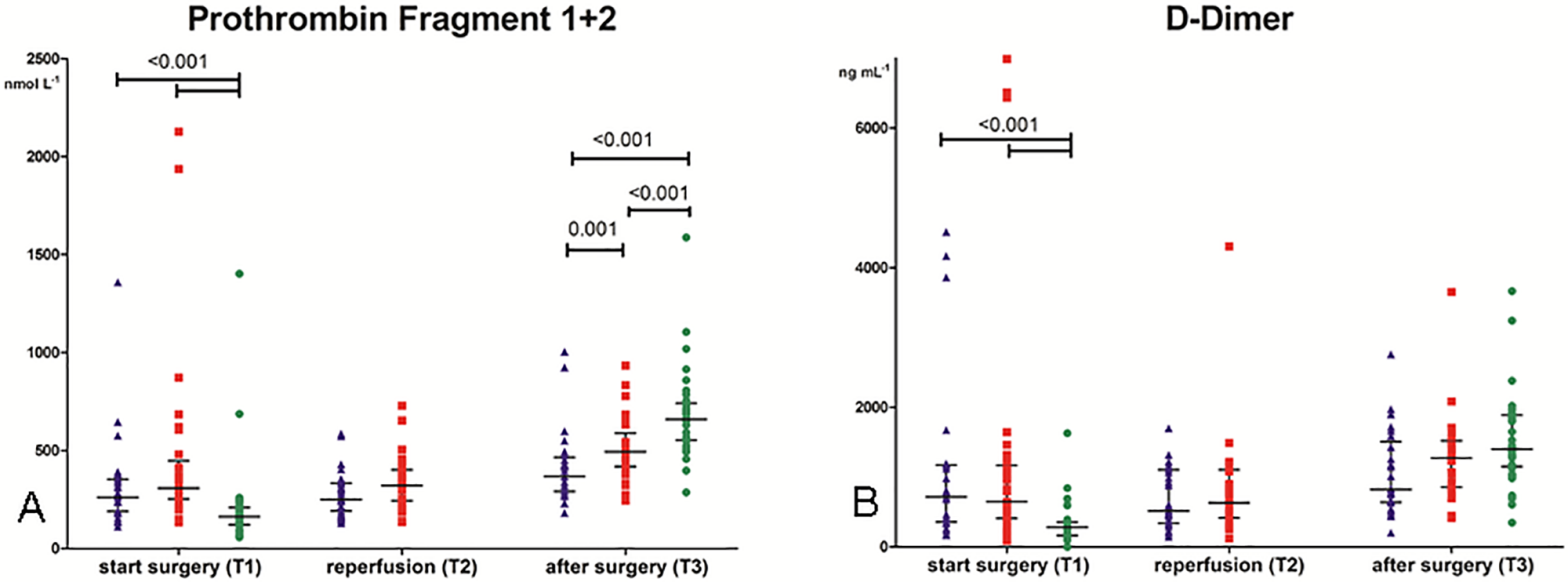
Markers of coagulation activation. Fig 3A: Levels of prothrombin fragment 1+2. Preemptive group (blue triangle), non-preemptive group (red square) and donors (green dot) before incision (T1), 5 minutes after reperfusion (T2) and 2 hours after surgery (T3). Data are given as medians with IQR. Fig 3B: Levels of D-dimer. Preemptive group (blue triangle), non-preemptive group (red square) and donors (green dot) before incision (T1), 5 minutes after reperfusion (T2) and 2 hours after surgery (T3). Data are given as medians with IQR.

Levels of markers of coagulation activation are shown in Fig 3A (F1+2) and Fig 3B (D-dimer). At baseline, levels of F1+2 were comparable between the preemptively and non-preemptively transplanted patients but higher compared to their donors (262 (190–353) and 309 (254–448) *versus* 163 (122–210) nmol L^-1^; P<0.001). After reperfusion, levels in the non-preemptive group showed a tendency to higher levels compared to the preemptive group, however this was not significant (322 (245–402) *versus* 251 (192–335) nmol L^-1^; P = 0.060). Post-operative F1+2 levels were higher in the non-preemptive group compared to the preemptive group but levels in both groups were lower compared to the donors (495 (419–589) and 368 (293–465) *versus* 660 (554–741) nmol L^-1^; P = 0.008 (non-preemptive vs preemptive), P<0.001 (non-preemptive vs donors) and P<0.001 (preemptive vs donors) (Fig 3A).

At baseline, the preemptive and the non-preemptive group showed similar levels of D-dimer. These levels were higher than levels in their donors (718 (359–1172) and 650 (410–1169) *versus* 283 (165–354) ng mL^-1^; P<0.001). Post-operatively, D-dimer levels in the non-preemptive group and the donors had increased, whereas levels in the preemptive group had not. At this time point, differences between groups were not significant (Fig 3B).

### Von Willebrand Factor

**Fig 4:**
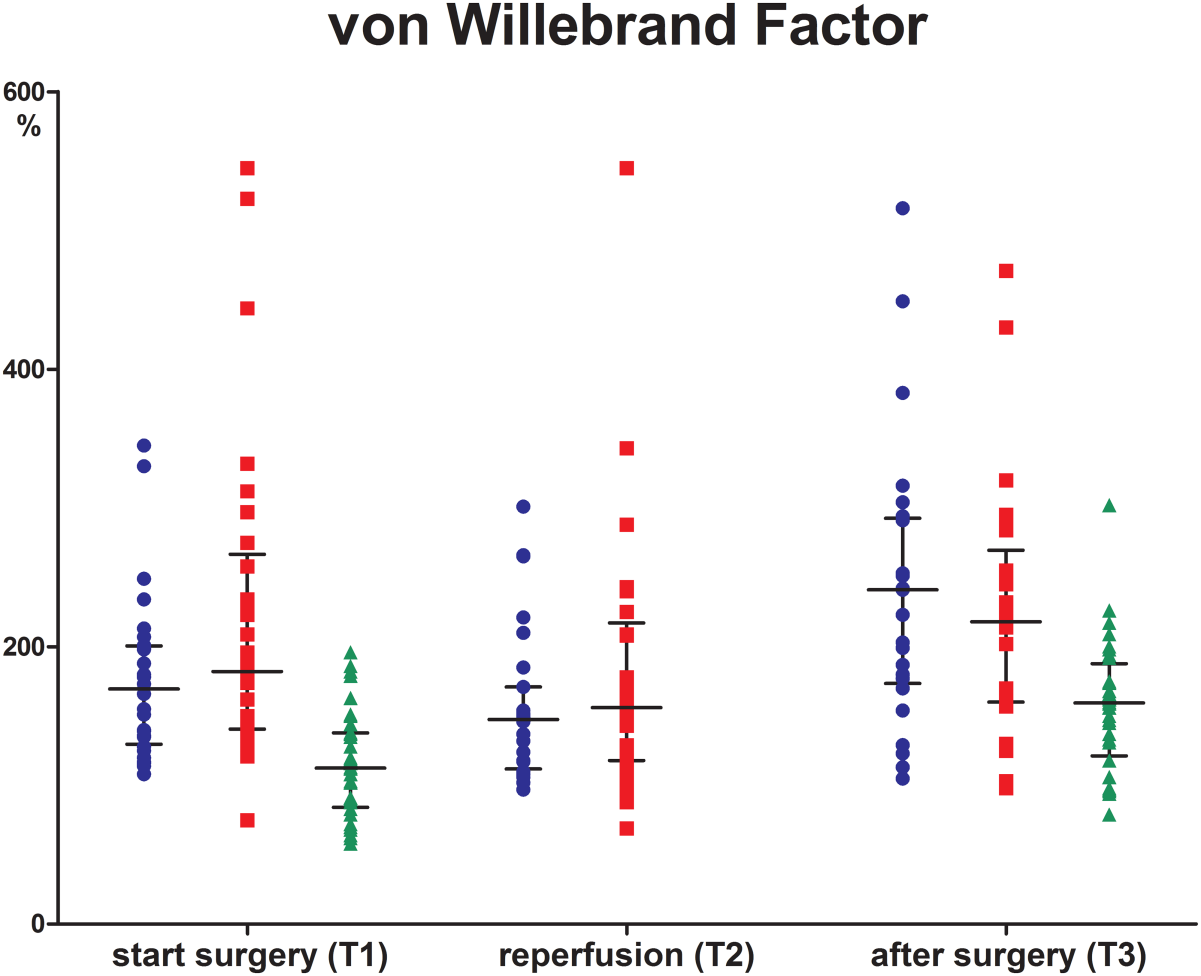
Von Willebrand Factor. Preemptive group (blue triangle), non-preemptive group (red square) and donors (green dot) before incision (T1), 5 minutes after reperfusion (T2) and 2 hours after surgery (T3). Data are given as medians with IQR.

Levels of VWF were similar between the preemptive and non-preemptive group at all time points. At T1 and T3 levels in both groups were higher compared to their donors; T1 (169% and 182% versus 113%, P<0.001 for both comparisons) and T3 (241 and 218 versus 160%, P<0.001)(Fig 4).

### TGA

**Fig 5:**
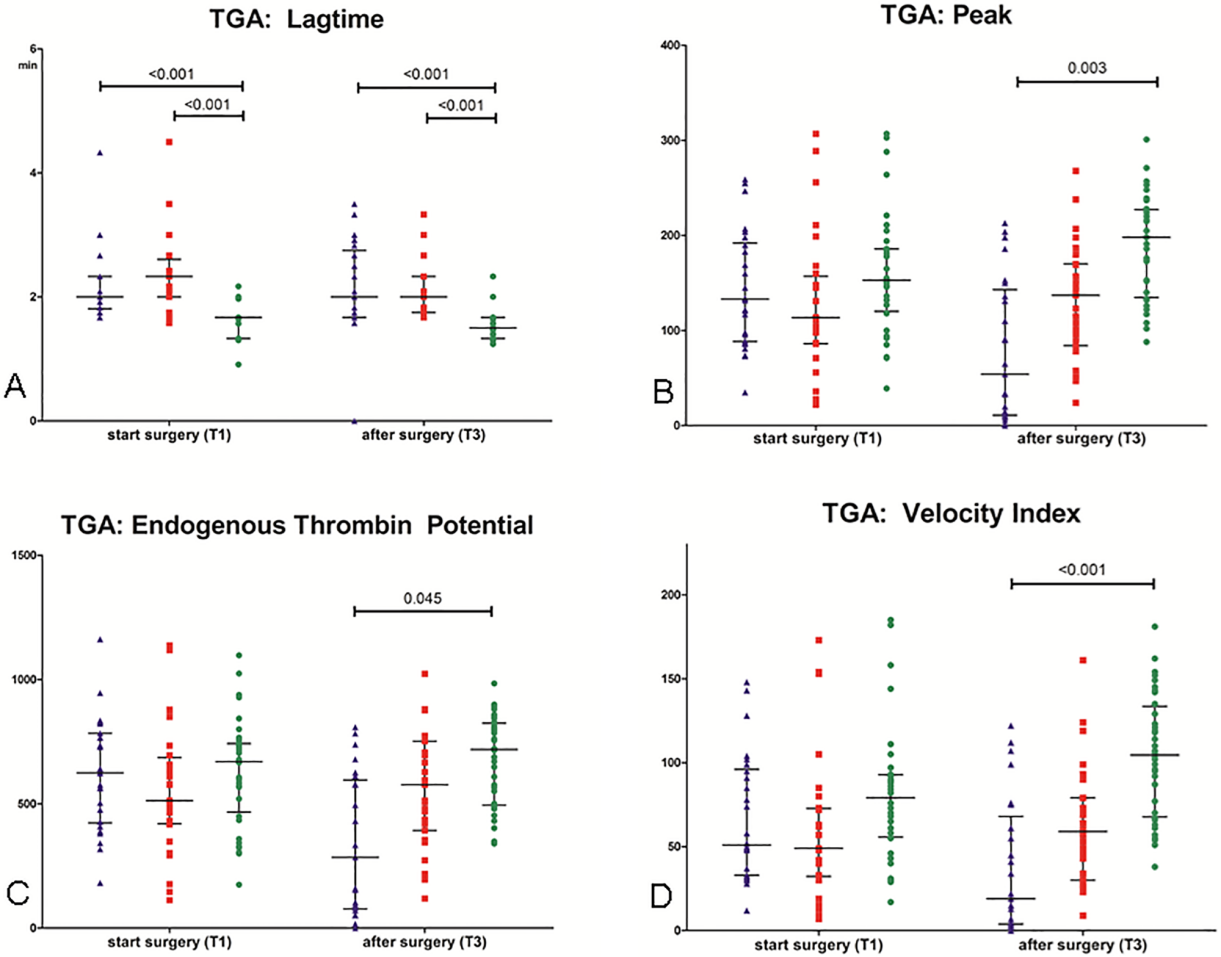
Thrombin generation assays. Fig 5A. TGA lagtime (A). Preemptive group (blue triangle), non-preemptive group (red square) and donors (green dot) before incision (T1) and 2 hours after surgery (T3). Data are given as medians with IQR. Fig 5B. TGA peak. Preemptive group (blue triangle), non-preemptive group (red square) and donors (green dot) before incision (T1) and 2 hours after surgery (T3). Data are given as medians with IQR. Fig 5C. TGA endogenous thrombin potential (ETP). Preemptive group (blue triangle), non-preemptive group (red square) and donors (green dot) before incision (T1) and 2 hours after surgery (T3). Data are given as medians with IQR. Fig 5D: TGA velocity index. Preemptive group (blue triangle), non-preemptive group (red square) and donors (green dot) before incision (T1) and 2 hours after surgery (T3). Data are given as medians with IQR.

Results of the thrombin generation assays are shown in Fig 5. At baseline, peak thrombin, ETP, and velocity index were comparable between the three groups. The lagtime was longer in the preemptive and non-preemptive group compared to their donors. Post-operative, peak thrombin, ETP, and velocity index were lower in the preemptive group compared to the donor group, and the lagtime was longer in the preemptive and non-preemptive group compared to the donor group.

### Fibrinolytic potential

**Fig 6:**
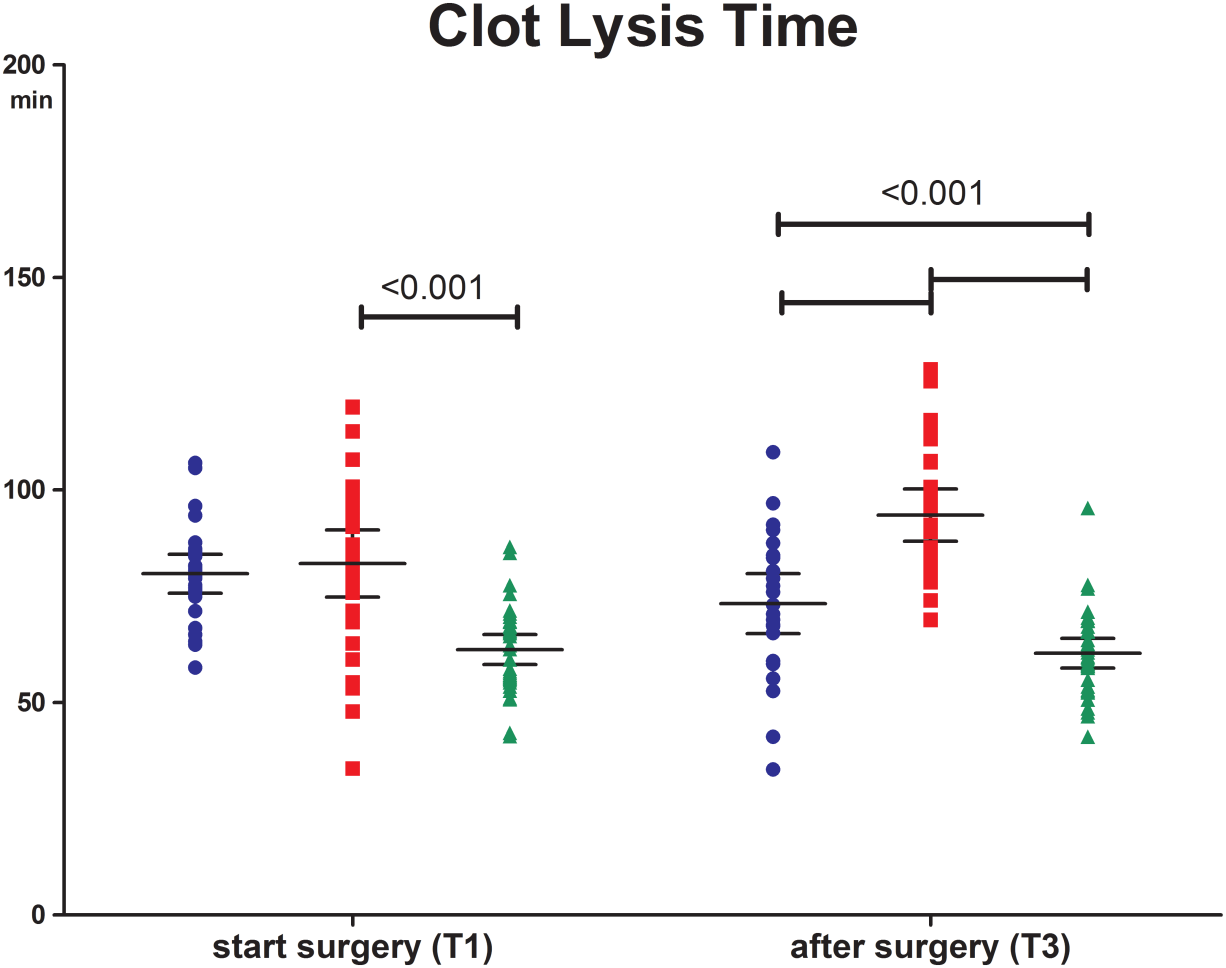
Clot lysis time. Preemptive group (blue triangle), non-preemptive group (red square) and donors (green dot) before incision (T1) and 2 hours after surgery (T3). Data are given as medians with IQR.

At baseline fibrinolytic potential, represented by CLT, was comparable between the preemptive and non-preemptive group. At this time point both groups showed a longer CLT compared to the donor group (80 (72–85) and 82 (70–97) versus 63 (56–69) min; P<0.0001). Post-operative, CLT in the non-preemptive group was longer compared to the preemptive and the donor group (92 (81–107) versus 73 (63–86) and 61 (54–67) min; P<0.0001) (Fig 6).

### Haemodialysis vs peritoneal dialysis

An additional analysis was performed within in the non-preemptive group comparing haemodialysis and peritoneal dialysis patients. There were no differences in haemostatic and fibrinolytic parameters between the two dialysis modalities. (S1–S4 Figs)

### Pathology

**Fig 7:**
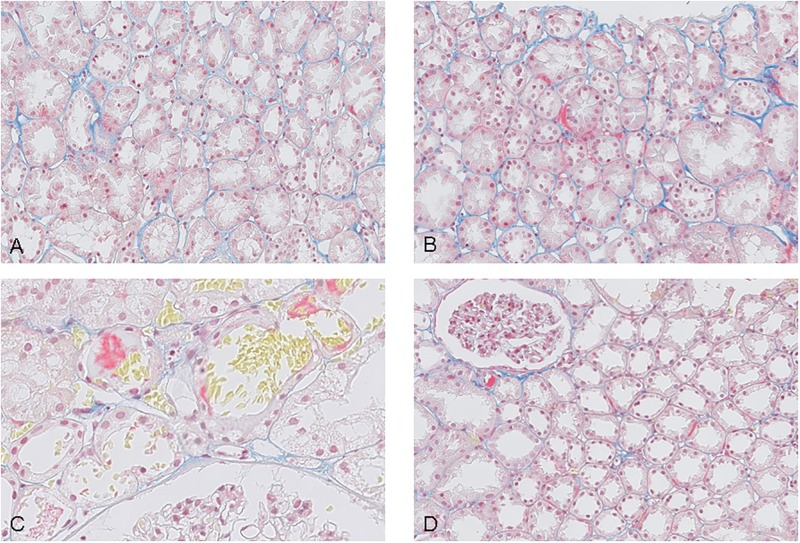
Martius Scarlet Blue stained reperfusion biopsies. Fibrin stains red, collagen blue. Fig 7A reperfusion biopsy negative for fibrin. Fig 7B reperfusion biopsy positive for fibrin Fig 7C reperfusion biopsy positive for fibrin Fig 7D reperfusion biopsy positive for fibrin.

A total of 38 reperfusion biopsy specimens were available. These were stained with Martius Scarlet Blue (fibrin stains red, collagen blue) and scored. Twelve biopsies consisted of renal medulla without glomeruli and were excluded. Of the 26 remaining biopsies 14 were obtained from preemptively transplanted patients and 12 from non-preemptively transplanted patients. In 6 biopsies focal discrete deposition of fibrin was seen in peritubular capillaries. Of these 6 positive biopsies, 4 patients were transplanted preemptively (29%) and 2 patients non-preemptively (17%), P 0,6522. (Fig 7)

## Discussion

This study demonstrates that prior to transplantation, preemptively and non-preemptively transplanted patients show a comparable hypercoagulable state as evidenced by both functional hemostatic tests and markers of in vivo activation of hemostasis compared to relatively healthy kidney donors. Therefore, the use of intraoperative anticoagulation solely in preemptively transplanted patients, as performed in our centre, does not appear justified.

Chronic kidney disease has been shown to be an independent risk factor for venous thromboembolisms (VTE, deep vein thrombosis and/or pulmonary embolism (PE)) in several cohort analysis and case control studies. In the Longitudinal Investigation of Thromboembolism Etiology (LITE) study in patients > 45 years, an eGFR between 15–60 ml min^-1^ was associated with a relative risk of VTE of 2.1 (95% CI 1.5–3.0). After adjustment for cardiovascular disease risk factors an increased risk was still observed with an adjusted relative risk of VTE of 1.7 (95% CI 1.2–2.5) compared to individuals with a normal kidney function [14]. In the PREVEND study the hazard ratio of VTE in patients with an eGFR between 30–60 ml min^-1^ was 1.6 (95% CI 0.9–2.8) and increased to 3.0 (95% CI 1.4–6.5) in the presence of albuminuria [15]. In a case-control study (the MEGA study) an eGFR 30–60 mL min^-1^ was associated with a 2.5-fold increased risk of VTE and an eGFR < 30 mL min^-1^ with a 5.5-fold increased risk compared with patients with normal kidney function (eGFR > 90 mL min^-1^) [16]. In this study the risk of VTE was additionally increased in combination with arterial thrombosis (odds ratio (OR), 4.9; 95% CI, 2.2–10.9), malignancy (OR 5.8; 95% CI, 2.8–12.1), surgery (OR 14.0; 95%, CI 5.0–39.4), immobilization (OR 17.1; 95% CI, 6.8–43.0) or thrombophilia (OR 17.8; 95% CI 4.0–78.7), with particularly high risks when three or more risk factors were present (OR 56.3; 95% CI, 7.6–419.3).

Overall these studies show that impaired kidney function is an independent risk factor for VTE and that this risk increases with decreasing eGFR and presence of additional risk factors for VTE such as immobilization and surgery. These studies, however, do not take dialysis into account. Previously it has been suggested that dialysis patients have lower mortality rates from VTE due to platelet dysfunction and bleeding tendency [17,18]. Recently a cohort analysis of 130 439 dialysis patients registered in the ERA-EDTA (European Renal Association-European Dialysis and Transplant Association) was performed with a median follow up period of 2 years. In contrast to former belief this analysis shows an unexpectedly high mortality rate from PE in dialysis patients namely 12.2 (95% CI 10.2–14.6) times higher than in general population. For myocardial infarction the mortality rate was 11.0 (95% CI 10.6–11.4) times higher and for stroke 8.4 (95% CI 8.0–8.8) times higher than in general population [19]. The authors did not find an association between mortality from pulmonary embolism and treatment modality (HD or PD). Wang and colleagues looked at the risk of PE among 106 231 Asian dialysis patients and found a nearly 3 times higher incidence of PE in dialysis patients compared to their matching control group without kidney disease with an adjusted hazard ratio of 2.0 (95% CI 1.6–2.5) [20]. In their analysis they performed a propensity score matched analysis of HD and PD treated patients and found that the PE incidence was higher in HD patients than in PD patients with an adjusted hazard ratio of 2.3 (95% CI 1.2–4.3). Furthermore the 30-day mortality from PE was higher in dialysis patients compared to their matching controls with an adjusted odds ratio of 2.6 (95% CI 1.3–5.0). These 2 large cohort studies suggest that the increased risk at VTE seen in patients with end stage renal disease (ESRD) is not rescinded by dialysis. Also after transplantation the incidence of VTE in the recipients is higher than in the general population. Incidences between 1% and 24% in the kidney transplant population are reported, compared to 8 to 27 per 10.000 person-years in the general population [21]. In a recent cohort analysis of 4,343 kidney transplant recipients, 8.9% of the patients developed a VTE during a median follow up period of 5.2 years (IQR 2.8–7.9) compared to 1.5% in the matched general population (17,372 members), HR 7.1 (95% CI 6.0–8.4). The highest incidence was found in the first 3 months after transplantation, of which the highest rate was in the early postoperative period, but remained elevated > 36 months post transplantation compared to the general population. The risk of death in recipients who experienced a VTE was 4 times higher compared to recipients without VTE and the death censored graft loss was 2 times higher [22]. In this analysis there was no difference in the incidence of VTE between preemptively and non-preemptively transplanted patients. Reported risk factors are donor specific (deceased donor), organ specific (longer cold ischemia time) and recipient specific (older age, history of hypercoagulabilty, underlying renal disease, type of immunosuppressive drugs, cytomegalovirus infection, cardiovascular disease and trauma, hospitalization and/or surgery)[21–23].

The balance between activation of the coagulation cascade and platelets on one hand and endogenous anticoagulant mechanisms on the other, prevents bleeding or formation of thromboembolisms under non-pathological conditions. ESRD, HD and PD may disturb this balance on many levels leading to a more pro- or anticoagulant state in the individual patient [9]. Furthermore, underlying diseases or inherited coagulation disorders may also influence the haemostatic state of renal transplant recipients making it even more difficult to identify patients at risk for graft thrombosis.

Unfortunately, to date there is no single accepted test to measure the global haemostatic state in an individual patient that allows us to predict the chance at bleeding or thromboembolic complications.

Therefore in this analysis we chose to asses individual components of haemostasis (primary, secondary and tertiary haemostasis) individually through 2 fundamentally different approaches (i.e., markers of in vivo activation and ex vivo “potential’ studies). The combined results of these tests gives a comprehensive picture of what is going on in the patient. We demonstrated that preemptive and non-preemptive patients have a comparable preoperative hypercoagulable state as assessed by PF4, F1+2, and D-dimer levels, Elevation of these markers indicate enhanced in vivo activation of platelets (PF4) and the coagulation system (F1+2, D-dimer). Furthermore, preoperative VWF levels were elevated in both preemptive and non-preemptive patients indicating endothelial activation. It has been well established that elevated plasma levels of VWF are associated with thrombotic risk in the general population [24]. In addition, we measured the capacity of patient plasma to generate thrombin after in vitro activation of coagulation using the thrombin generation test, and assessed the capacity of an in vitro formed clot to be broken down by the fibrinolytic system (CLT). These two tests indicate the capacity of the coagulation and fibrinolytic system to respond to injury (haemostatic ‘potential’). The CLT was elevated in both recipient groups. Our group has previously shown that elevated CLT values are associated with an increased risk for both venous and arterial events in the general population [25]. A limitation in our analysis is that we did not asses platelet function. Assessing platelet function by suspension aggregometry, for example, would have required immediate analyses in whole blood, whereas all other tests were performed in stored plasma samples. Logistically, immediate analyses of platelet function were challenging, which is why we chose not to include this particular test. Nevertheless, we do feel that the PF4 and sPselectin measurements do capture in vivo platelet activation.

Because of small numbers, patients treated with HD and PD were pooled in one group of non-preemptively transplanted patients which might have led to a potential bias. A history of PD has been shown to be an independent risk factor for renal graft thrombosis in several retrospective studies [4, 26, 27]. In their database analysis of the United Network for Organ Sharing (UNOS) of 84.513 kidney transplant procedures, Ojo and colleagues reported a general incidence of renal vein thrombosis (RVT) of 0.8% and in a selected patient group (n = 2223) an odds ratio of RVT of 1.9 (95% CI 1.3–2.7) in PD compared to HD patients [4]. Underlying mechanisms may be an increase in thrombogenic proteins like apolipoprotein (a), plasminogen activator inhibitor type 1 (PAI-1, inhibits activation of fibrinolysis) and fibrinogen, increased levels of coagulation factors (II, VII, VIII, IX, X, XI and XII) and increased platelet count, seen in PD treated patients[28–30]. Also patient selection may be a contributing factor in this increased thrombotic risk since patients on HD are sometimes switched to PD because of vascular access problems due to thrombotic events. This switch has been shown to be an independent predictor of graft thrombosis with an OR of RVT of 3.6 (95% CI 2.7–5.5) in case of patients switched from HD to PD [4]. However, several smaller studies (n<1000 patients) did not find a difference in the incidence of graft thrombosis between PD or HD treated patients [3,31, 32]. We looked at the two dialysis modalities as separate groups and did not find a difference in haemostatic or fibrinolytic parameters tested between PD and HD.

Post-operatively, non-preemptively transplanted patients displayed a relative hypercoagulable profile compared to pre-emptively transplanted patients as evidenced by increased levels of F1+2 and d-dimer, increased thrombin generation, and increased CLT. The use of heparin in preemptively transplanted patients might have influenced some of the measurements on T2 and T3 in this group. Decreased levels of PF4 in the preemptive group compared to the non-preemptive group, might be a laboratory artefact since the ELISA used does not recognise PF4-heparin complexes. Furthermore, reduced levels of prothrombin fragment 1+2 are most likely due to inhibition of thrombin generation by heparin. In the non-preemptive group and the donors, levels of F1+2 and D-dimer at T3 are increased postoperatively compared to preoperative levels. Activation of the coagulation system after surgery due to a combination of surgical injury and systemic inflammatory responses has been well described [24–26]. Levels of F1+2, fibrinogen and D-dimer can remain elevated up to one month after surgery [27].

In our population, the postoperative increase in F1+2 and D-dimer was most apparent in the donors, which in contrast to the recipients, underwent a laparoscopic procedure. Studies comparing open and laparoscopic cholecystectomy reported a higher level of activation in the open procedures, which is thought to be related to a higher degree of tissue injury in the open procedures [33,34]. In studies comparing laparoscopic cholecystectomy to less invasive open procedures such as open hernia repair, no difference was seen [35,36]. However, in our population, the open extra-peritoneal kidney transplantation might in fact be less invasive compared to a laparoscopic trans-peritoneal nephrectomy explaining lower levels of markers of coagulation activation in the open procedure. The use of immunosuppressive induction therapy, consisting of methylprednisolone and basiliximab, in recipients could also have led to a suppression of systemic inflammatory response with less activation of the haemostatic system. Another explanation could be that levels of F1+2 and D-dimer in recipients increased on a later time point than T3 (2 hours after skin closure). Since the primary focus of this study was to assess whether obvious differences in haemostatic status between pre-emptively and non-preemptively transplanted patients were present pretransplantation we did not include sample points beyond 2 hours after skin closure. We are therefore unaware of the expression profile of coagulation markers beyond this time point.

Whether recipients benefit from a single intraoperative dose of 5000 IU unfractionated heparin combined with routine thromboprophylaxis with LMWH once daily postoperative (as performed in our centre) is unclear. No differences were seen in formation of microthrombi in the MSB stained biopsies between the preemptive group (treated with heparin) and non-preemptive group (no-heparin).

Ng and co-workers evaluated several heparin anticoagulation protocols in the postoperative period after kidney transplantation [37]. They concluded that the prophylactic use of heparin (5000 IU UFH sc twice daily) is safe. The incidence of major bleeding complications was comparable between the prophylactic and the no-heparin group. In contrast, therapeutic use of heparin (IV, target aPTT 50–120 s) was associated with an increase in postoperative major bleeding episodes. This has been confirmed in other studies evaluating therapeutic use of heparin in high risk kidney transplant patients [38–39]. Regarding effectiveness, the rate of thrombosis was highest in the no-heparin group (1.1%) compared to prophylactic (0.4%) or therapeutic (0.0%) heparin group.

Interestingly, a recent review reports the use of heparin and heparinoids as inhibitors of the complement system [40]. Activation of the complement system plays an important role in graft rejection and ischemia and reperfusion injury. The authors suggest a potential role of the use of heparin in modification of this complement activation. The complement system, the coagulation cascade and the fibrinolysis cascade crosstalk through many direct and indirect interactions. Thrombin and plasmin directly cleave component C3, as well as its activation fragments. Furthermore thrombin can cleave C5 into C5a independently of C3 [41]. Inhibition of thrombin formation by heparin might be a potential pathway to inhibit complement activation. Timing of administration and dosage however is not clear and more research has to be performed to study this potential application of heparin.

We did not perform a power calculation, which would not have been possible as we did not have data on the variation of the various haemostatic tests performed in this particular patient population, and had no clear indications as to which differences would be clinically relevant. The aim of the study was to see whether there would be differences between the preemptive and non-preemptive groups, and if so, to obtain an estimate of the size of the difference. Although our results would benefit from confirmation in an independent study, the absence of a clear difference at baseline between the groups justifies our conclusion that our differential heparin policy should be questioned, and does not justify larger, powered follow-up studies to get an exact number of the effect size.

In conclusion, our results indicate that in contrast to common clinical belief, the haemostatic state in preemptively and non-preemptively transplanted patients is comparable prior to transplantation, and that both groups show a preoperative hypercoagulable state compared to their living kidney donors. Whether these pre-emptive recipients benefit from a single dose of 5000 IU UFH intraoperatively is unclear, but based on our results a distinction in intraoperative heparin administration between preemptive or non-preemptive transplantation does not seem justified.

## Supporting information

S1 Table Correlation of preoperative urea levels (mmol L^-1^) with the haemostatic and fibrinolytic parameters measured at sample point T1, r = Pearsons correlation coefficient. (DOCX)

S1 Fig Markers of platelet activation in patients treated with haemodialysis vs. peritoneal dialysis. Part A. Levels of platelet factor 4 in patients treated with haemodialysis (blue dots) and patients treated with peritoneal dialysis (red squares). Before incision (T1), 5 minutes after reperfusion (T2) and 2 hours after surgery (T3). Data are given as medians with IQR. Part B. Levels of soluble P-selectin in patients treated with haemodialysis (blue dots) and patients treated with peritoneal dialysis (red squares). Before incision (T1), 5 minutes after reperfusion (T2) and 2 hours after surgery (T3). Data are given as medians with IQR.
(TIF)

S2 Fig Markers of coagulation activation in patients treated with haemodialysis vs. peritoneal dialysis. Part A. Levels of prothrombin fragment 1+2 in patients treated with haemodialysis (blue dots) and patients treated with peritoneal dialysis (red squares). Before incision (T1), 5 minutes after reperfusion (T2) and 2 hours after surgery (T3). Data are given as medians with IQR. Part B. Levels of D-dimer in patients treated with haemodialysis (blue dots) and patients treated with peritoneal dialysis (red squares). Before incision (T1), 5 minutes after reperfusion (T2) and 2 hours after surgery (T3). Data are given as medians with IQR.
(TIF)

S3 Fig Von Willebrand Factor in patients treated with haemodialysis vs. peritoneal dialysis. Level of Von Willebrand Factor in patients treated with haemodialysis (blue dots) and patients treated with peritoneal dialysis (red squares). Before incision (T1), 5 minutes after reperfusion (T2) and 2 hours after surgery (T3). Data are given as medians with IQR.
(TIF)

S4 Fig Clot lysis time in patients treated with haemodialysis vs. peritoneal dialysis. Clot Lysis Time in patients treated with haemodialysis (blue dots) and patients treated with peritoneal dialysis (red squares). Before incision (T1) and 2 hours after surgery (T3). Data are given as medians with IQR.
(TIF)
