# Supplementary material for: Preemptively and non-preemptively transplanted patients show a comparable hypercoagulable state prior to kidney transplantation compared to living kidney donors
Source: PLoS One. 2018 Jul 16;13(7):e0200537. doi: 10.1371/journal.pone.0200537 (PMC6047796; doi:10.1371/journal.pone.0200537)
Supplement: S1 Table — (DOCX) [file pone.0200537.s001.docx]

S1 table: Correlation of preoperative urea levels (mmol L-1 ) with the haemostatic and fibrinolytic parameters measured at sample point T1, r= Pearsons correlation coefficient

|  | Urea pre-transplantation  Correlation r P-value | |
| --- | --- | --- |
| Platelet factor 4 | 0.1457 | 0.2840 |
| sPselectin | 0.0540 | 0.6927 |
| F1+2 | 0.1799 | 0.1887 |
| D-dimer | 0.5220 | <0.0001 |
| Von Willebrand Factor | -0.0460 | 0.7364 |
| TGA lagtime | 0.1131 | 06163 |
| TGA peak | 0.2232 | 0.3180 |
| TGA ETP | 0.2256 | 0.3128 |
| TGA velocity index | 0.2165 | 0.3331 |
| CLT | 0.0188 | 0.8919 |
