# Supplementary figures and images for: Preemptively and non-preemptively transplanted patients show a comparable hypercoagulable state prior to kidney transplantation compared to living kidney donors
Source: PLoS One. 2018 Jul 16;13(7):e0200537. doi: 10.1371/journal.pone.0200537 (PMC6047796; doi:10.1371/journal.pone.0200537)

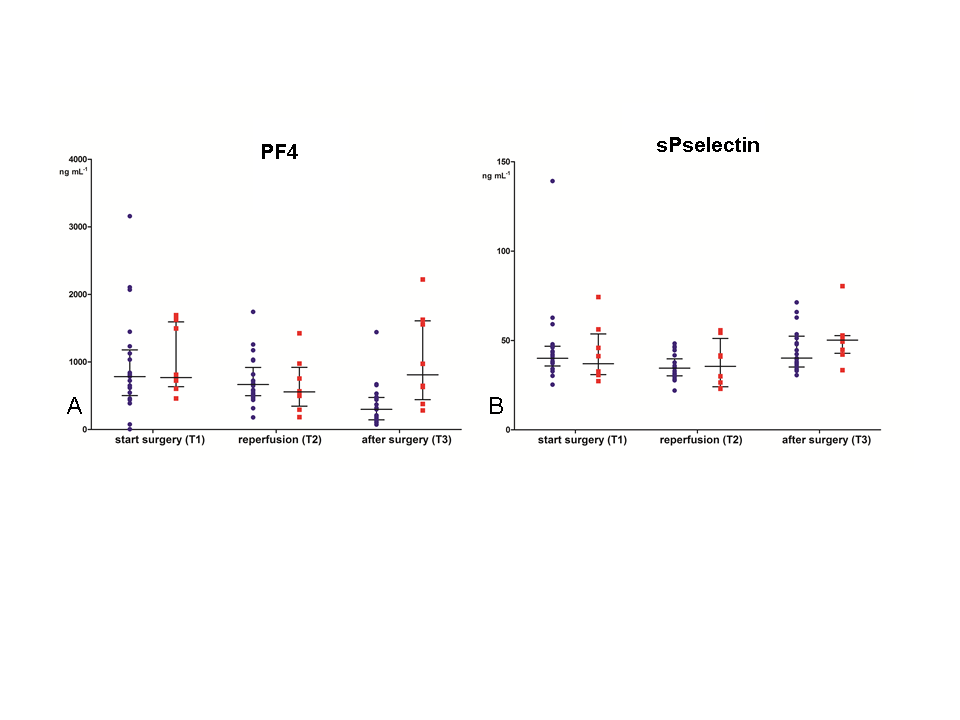

Supplement: S1 Fig — Part A. Levels of platelet factor 4 in patients treated with haemodialysis (blue dots) and patients treated with peritoneal dialysis (red squares). Before incision (T1), 5 minutes after reperfusion (T2) and 2 hours after surgery (T3). Data are given as medians with IQR. Part B. Levels of soluble P-selectin in patients treated with haemodialysis (blue dots) and patients treated with peritoneal dialysis (red squares). Before incision (T1), 5 minutes after reperfusion (T2) and 2 hours after surgery (T3). Data are given as medians with IQR. (TIF) [file pone.0200537.s002.tif]

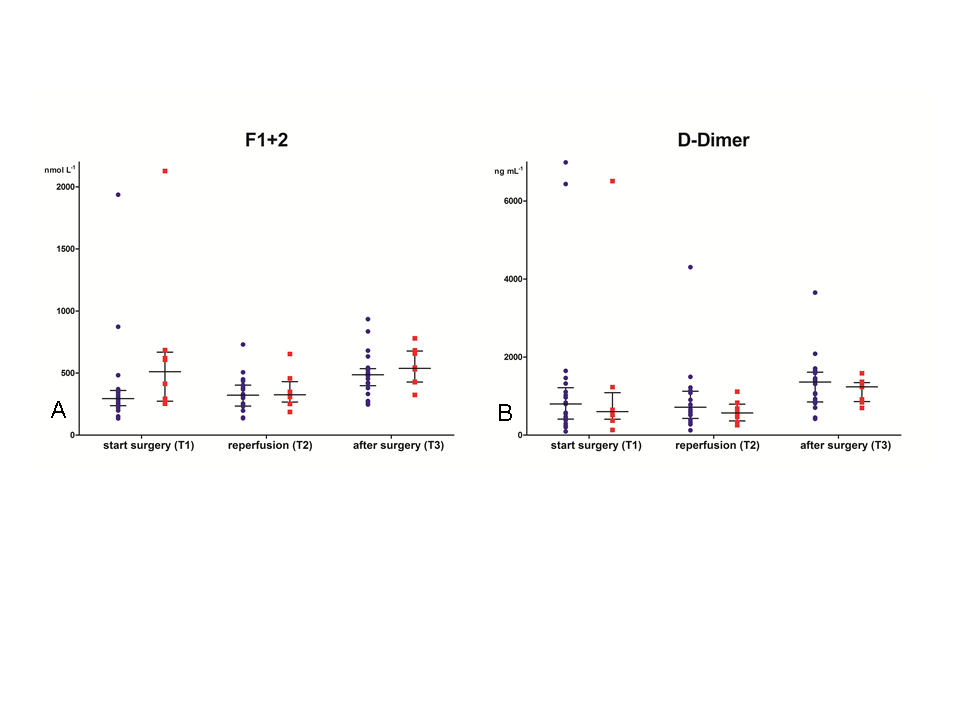

Supplement: S2 Fig — Part A. Levels of prothrombin fragment 1+2 in patients treated with haemodialysis (blue dots) and patients treated with peritoneal dialysis (red squares). Before incision (T1), 5 minutes after reperfusion (T2) and 2 hours after surgery (T3). Data are given as medians with IQR. Part B. Levels of D-dimer in patients treated with haemodialysis (blue dots) and patients treated with peritoneal dialysis (red squares). Before incision (T1), 5 minutes after reperfusion (T2) and 2 hours after surgery (T3). Data are given as medians with IQR. (TIF) [file pone.0200537.s003.tif]
